# Impact of Inspiratory Muscle Training in Individuals with Gastroesophageal Reflux Disease: A Randomized Controlled Trial Protocol

**DOI:** 10.3390/mps9020032

**Published:** 2026-02-27

**Authors:** Stylianos Syropoulos, Maria Moutzouri, Eirini Grammatopoulou, Irini Patsaki

**Affiliations:** Laboratory of Advanced Physiotherapy, Department of Physiotherapy, University of West Attica, 122 43 Athens, Greece; ssyropoulos@uniwa.gr (S.S.); moutzouri@uniwa.gr (M.M.);

**Keywords:** inspiratory muscle exercises, gastroesophageal reflux disorder, diaphragmatic function, maximal inspiratory strength

## Abstract

Gastroesophageal reflux disease (GERD) is a common chronic condition mainly caused by the dysfunction of the antireflux mechanism at the gastroesophageal junction. This is composed of the lower esophageal sphincter and the crural diaphragm. Increasing evidence suggests that diaphragmatic dysfunction and reduced inspiratory muscle strength may contribute to the persistence of GERD symptoms. Although respiratory physiotherapy has shown beneficial effects, the role of a structured inspiratory muscle training (IMT) program has not been sufficiently examined. This study aims to investigate the effects of an inspiratory muscle training program on inspiratory muscle strength and secondary clinical outcomes in individuals with GERD. A total of thirty adults with a confirmed GERD diagnosis will be enrolled in a two-arm randomized controlled trial. These volunteers will be randomly assigned either to the experimental group, which will undergo a 3-month inspiratory muscle training (IMT) using tapered flow resistive loading at 40% of maximal inspiratory pressure (MIP), or to the control group, which will receive sham IMT with a consistent low resistance. Primary outcomes will include maximal inspiratory pressure (MIP) and maximal dynamic inspiratory pressure (S-index). Secondary outcomes will assess GERD symptoms, disease-related quality of life, and pulmonary function. Measurements will be performed at baseline, at three months of intervention, and at six months from recruitment (follow-up). IMT is expected to lead to significant improvements in inspiratory muscle strength, symptom burden, and quality of life compared with sham training. This trial will provide novel evidence regarding the role of inspiratory muscle training as a non-pharmacological intervention in the management of GERD. **Trial registration:** ClinicalTrials.gov Identifier: NCT07131397.

## 1. Introduction

Gastroesophageal reflux disease (GERD) is a chronic condition of the upper gastrointestinal tract, characterized by the repeated backward flow of stomach contents into the esophagus, leading to esophageal symptoms such as heartburn, as well as extraesophageal ones like chronic cough and wheezing. The persistence of these symptoms is consistent with an impaired antireflux barrier [1].

The antireflux barrier is a functional unit consisting of the intrinsic lower esophageal sphincter (LES) and the diaphragmatic sphincter [2]. During normal processes such as swallowing, a coordinated relaxation of both components allows the passage of the bolus into the stomach [3,4]. This is followed by a rapid restoration of the barrier pressure, which is higher than the intra-abdominal pressure, preventing the reflux of gastric contents. Transient lower esophageal sphincter relaxations represent another important contributor to reflux events. Although this phenomenon is physiological, its increased frequency and prolonged duration in individuals with GERD result in repeated inhibition of diaphragmatic activity, thereby weakening the extrinsic component of the antireflux barrier [3].

In GERD, the coordinated activation of the sphincters is often disrupted, leading to increased LES and femoral septum distensibility, thus reducing resistance to reflux episodes [3,4]. These episodes allow hydrochloric acid, one of the most toxic components of gastric juice, to cause inflammation in the esophageal epithelium, leading to severe esophageal mucosal damage [5]. Abnormal changes in intrathoracic and intra-abdominal pressures that alter diaphragmatic movement and contractility may be harmful [6,7,8].

Increasing evidence suggests that GERD is not limited to the gastrointestinal tract but is also associated with respiratory deficiencies. Individuals with GERD, even in the absence of diagnosed pulmonary disease, have been reported to exhibit reduced inspiratory muscle strength, altered airflow dynamics, and obstructive or restrictive airway pathology [9,10,11]. Proposed mechanisms include microaspiration of gastric contents and reflex bronchoconstriction mediated via the pneumovagal tract [12]. These respiratory manifestations contribute significantly to symptom burden and reduced quality of life [13].

Non-pharmacological interventions targeting the physiological mechanism of the antireflux barrier, especially those that have been widely implemented in respiratory disorders, have therefore gained increasing attention [14]. Respiratory physiotherapy and specific interventions targeting the enhancement of diaphragmatic function have been found to alleviate reflux symptoms and improve patient-reported outcomes [15]. Additionally, inspiratory muscle training (IMT) represents a focused strategy to strengthen the diaphragm through externally imposed specific load related to the individual’s maximal inspiratory pressure [16,17]. This is an individualized intervention with preliminary studies suggesting that it could augment gastroesophageal junction competence and reduce symptom severity. Thus, medication and the possibility of surgery might be reduced [18]. However, evidence remains limited by heterogeneous methodologies and trials with small sample sizes [16].

The present randomized controlled trial protocol is designed to address these gaps by systematically evaluating the effects of a structured IMT program on inspiratory muscle strength and disease-specific patient-reported outcomes in people with GERD. This study aims to clarify the role of diaphragmatic strengthening as a non-pharmacological management of GERD.

## 2. Objectives

The main aim of this randomized controlled trial is to assess whether a structured inspiratory muscle training intervention enhances inspiratory muscle strength, assessed through maximal inspiratory pressure and maximal dynamic inspiratory pressure, in patients with gastroesophageal reflux disease. Secondary objectives include evaluating changes in GERD symptom severity, disease-related quality of life, and pulmonary function following the intervention.

## 3. Methods

### 3.1. Study Design

**Figure 1:**
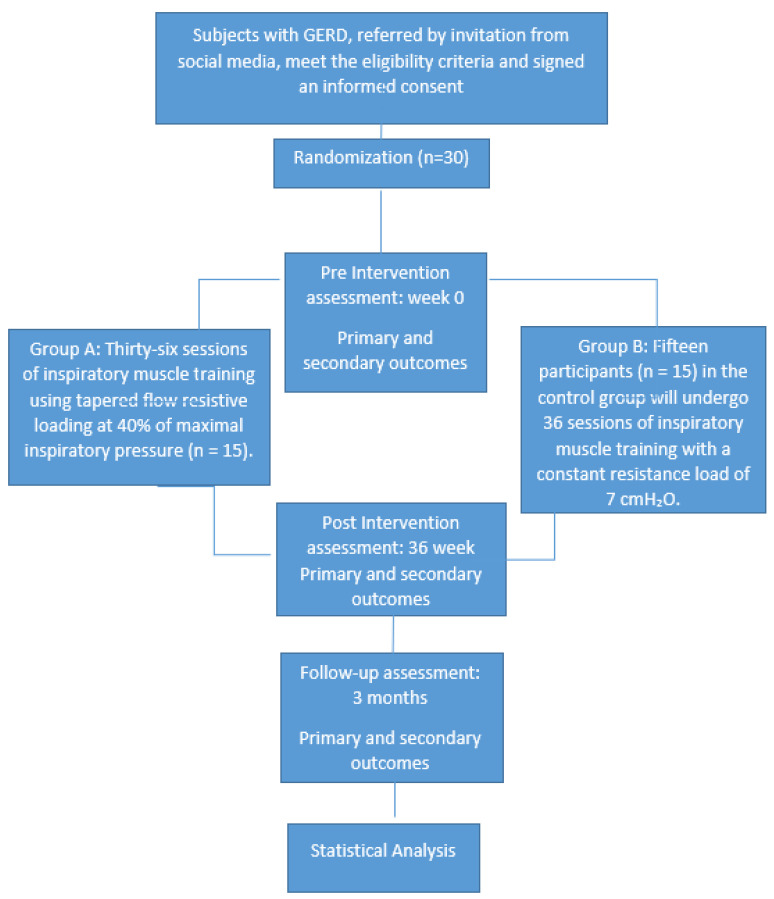
The design of the study is outlined in a flowchart prepared in line with the SPIRIT statement.

This study is structured as a two-arm, parallel-group randomized controlled trial, conducted in line with the CONSORT guidelines and the SPIRIT recommendations for interventional trial protocols [19]. The study’s design is presented in Figure 1. The protocol has been prospectively registered on ClinicalTrials.gov (NCT07131397).

### 3.2. Study Setting

The trial will be coordinated by the Laboratory of Advanced Physiotherapy, Department of Physiotherapy, University of West Attica. Participant recruitment, delivery of interventions, and outcome assessments will take place at a private physiotherapy clinic located in Athens, Greece.

### 3.3. Recruitment Procedures

Participants will be recruited via social media invitations, providing a medical diagnosis of GERD and spirometry by a gastroenterologist and pulmonologist, respectively.

Diagnosis of GERD should be in accordance with the Lyon consensus criteria [20]. Measurement of the spirometry and the S index and the following information will be explained to the participants: (a) objectives, methods and clinical significance of the study, (b) the right to withdraw from the study at any time, (c) the voluntary nature of their participation, (d) information about the results of their assessments and (e) that their choice to take part in the study will not influence the delivery of research-related services, and failure to attend more than two scheduled treatment sessions or any deterioration in GERD symptoms will result in withdrawal from the study. All participants will be required to provide written informed consent, signed by the participant, the principal investigator, and two witnesses. This consent will authorize participation in the study and the publication of results, while allowing participants the right to withdraw at any point.

### 3.4. Randomization and Blinding Procedures

The allocation concealment will be ensured through the randomization process. Sealed, opaque envelopes will be used, numbered consecutively by an Assistant Professor of the Laboratory of Advanced Physiotherapy, University of West Attica, who will not participate in any other process of the study. Participant assignment will be concealed from both participants and evaluators, while the principal investigator will be informed, in accordance with a single-blinding procedure.

### 3.5. Participants and Eligibility Criteria

Adults aged 18–70 years with a confirmed diagnosis of gastroesophageal reflux disease by a gastroenterologist are eligible to participate (inclusion criteria). Selection and initial assessment will be done by a physiotherapist [21,22]. Exclusion criteria include pregnancy, previous gastric or duodenal surgery, systemic connective tissue diseases, tuberculosis, and diagnosed mental disorders [23].

Participants’ characteristics: The sample will comprise participants of all genders, including both males and females with diagnosed GERD, from the Attica region. The minimum sample size was determined using the G*Power software (version 3.1.9.7), based on an effect size of 1.3 for MIP reported by Fonseca et al. (2014) [24]. Based on an effect size of 1.3, a statistical power of 0.80, and an α level of 0.05, the minimum required sample size was 22 participants. Accounting for a potential 15% dropout rate, the final minimum sample size was set at 25.

### 3.6. Outcome Measurements

The outcome assessments have been selected to capture both physiological changes in inspiratory muscle performance as well as clinically significant effects on the severity and frequency of GERD symptoms. A trained physiotherapist will perform: (a) MIP (Micro RPM), according to ERS statement [25,26], (b) S-index measurement (Powerbreathe K5) [27,28] and will administer the Reflux Disease Questionnaire [29,30,31] and Reflux-Qual Short Form Questionnaire [32,33]. All measurements will be performed in random order.

Respiratory function will be assessed with the measurement of FEV1/FVC% using the Spirolab iii spirometer (Medical International Research, Inc., Rome, Italy). This will be performed by a pulmonologist with adequate training.

All measurements will be performed at three pre-specified time points: at baseline (T1), immediately after completion of the 12-week intervention (T2), and at the six-month follow-up (T3) (Table 1).

#### 3.6.1. Primary Outcomes

Inspiratory muscle strength is the primary outcome of the study. Maximal inspiratory pressure (MIP) will be used as an indicator of overall inspiratory muscle strength, reflecting the maximum static pressure generated against an obstructed airway. This measure has been chosen for its clinical relevance in identifying diaphragmatic weakness and monitoring adaptations induced by training [34]. The ATS/ERS guidelines recommend performing 3 measurement attempts. According to them, an absolute maximum inspiratory pressure value at the mouthpiece of 80 cmH_2_O usually excludes clinically significant weakness of the inspiratory muscles. In older ages, the limit for men is 60 cm H_2_O and for women, it is 40 cmH_2_O [34]. The measurement of maximum expiratory pressure will be performed using the same equipment, measures the strength of the expiratory muscles and is performed with the patient in a sitting position and closed nose. A maximal inspiratory effort is commanded for 1–2 s and repeated 3–8 times. In women, the normal limit is 120 cmH_2_O of mercury, while in men, it is 150 cm of mercury [35]. In addition, maximal dynamic inspiratory pressure will be assessed using the S-index. The S-index is a new non-invasive method for the dynamic assessment of inspiratory muscle strength. The measurement is performed using an electronic inspiratory muscle training device that applies conical flow resistance as a loading method. The measurement includes 10 maneuvers, with the participant being encouraged to make a maximum effort up to the total lung capacity. At the beginning of the measurement, a warm-up session (one set of 30 breaths at 40% of S-index) has been included to enhance pulmonary function and attenuate the ‘learning effect’ of repeated measurements [27]. This outcome provides information on inspiratory muscle performance under conditions that more closely resemble functional breathing tasks and may be more sensitive to training-related changes than static measurements alone [27,36]. The average value of the S index in healthy populations is 159 cmH_2_O in men and 115.29 cmH_2_O in women [28].

#### 3.6.2. Secondary Outcomes

The severity of GERD symptoms will be assessed using the Reflux Disease Questionnaire (RDQ), a patient-reported outcome measure that records the frequency and intensity of symptoms such as retrosternal heartburn and pain, upper stomach heartburn and pain, sour taste in the mouth, and regurgitation, which are associated with central reflux. The RDQ has been selected based on dyspepsia guidelines for its established use in clinical research to aid in diagnosis (sensitivity 88.1% and specificity 29.8%) [31]. It has been previously validated in Greek-speaking populations with high overall internal consistency (alpha value: 0.91) on three factors related to heartburn, regurgitation, and dyspepsia [29].

Health-related quality of life, specifically related to GERD, will be assessed with the Short Form Regurgitation Quality Questionnaire (RQS) [33]. This tool assesses the impact of reflux symptoms on daily activities (4 questions), psychological well-being (2 questions), sleep and eating behavior (2 questions), thus providing an insight into the broader consequences of symptom change beyond physiological measures [33]. The questions are scored on a 5-point scale, and the mean value ranges from 0 (low) to 100 (high). Its validation in the Greek population has been performed by Georganta et al. 2002 showing high internal consistency and validity with that of the sph-36 questionnaire (r > 0.40, *p* < 0.05) [32].

Pulmonary function will be assessed using spirometry, with particular emphasis on the FEV1/FVC ratio, with the main aim of the study being to determine whether or not undiagnosed lung disease is present, expressed as a percentage of predicted values. This procedure will be conducted in accordance with the guidelines proposed by Knudson, Slatin, Lebowitz, and Burrows (1976) [37]. Internal validity will be ensured by calibrating the device before each measurement using a flow–volume syringe. The duration of each subject’s attempt will be 6 s and the best recording will be made after completing at least three acceptable attempts [37,38].

### 3.7. Interventions

An informative session on diaphragmatic breathing with simulated inspiratory muscle exercise after randomization will be given to participants of both groups. This will enhance the role of physiotherapy, specifically the development of a trusting relationship between therapist and participant, increasing their self-confidence [39]. The long-term goals of managing the underlying disease and the health belief model will determine the structure of the session [40].

Intervention group: Participants allocated to the intervention group will follow a 12-week inspiratory muscle training program consisting of three 15 min sessions per week in the morning or afternoon 2 h after breakfast or lunch [41]. Training will be performed using an electronic inspiratory muscle trainer that provides a tapered flow of load resistance, initially at 40% of each participant’s MIP performing 30 breaths per session with 1 set of 30 repetitions [24,42]. Resistance will be progressively increased on a weekly basis at a rate of 10% of the initial maximal inspiratory pressure measurement if patients at the end of the week experience difficulty 3–4 on the Borg scale [43]. Otherwise, the resistance will remain the same. Resistance will also be maintained if the participants present persistent reflux symptoms.

Control group: Participants assigned to the control group will perform sham inspiratory muscle training using the same device with a constant minimal resistance 7 cmH_2_O [23].

A training diary will be kept for all participants that will include all information on the progression of training, missed sessions (along with the reason) and monitoring of their symptoms and medication.

### 3.8. Methods of Data Collection

Data collection, both prior to and following the intervention, will be conducted by the principal researcher. The moderation of these procedures will be done by the main researchers’ supervising professor.

### 3.9. Data Management

Data will be gathered at predefined intervals under the supervision of a committee responsible for monitoring the data, comprised of members from the Department of Physiotherapy at UNIWA.

Physical records, including printed assessment forms and signed consent documents, will be securely maintained at the study site. All records will undergo monthly backups until the end of the trial to safeguard data integrity. Statistical analyses will be performed by the study statistician on the complete dataset once the study has concluded.

## 4. Analytical Approaches for Statistical Evaluation of the Data

Statistical analyses will be performed using the Statistical Package for the Social Sciences (SPSS, version 29; IBM Corp., Armonk, NY, USA). Prior to inferential analysis, data will be screened for completeness and distributional characteristics. The normality of continuous variables will be examined using the Shapiro–Wilk test. Descriptive statistics will be used to present and summarize participants’ characteristics.

To evaluate the reliability of repeated measurements across the assessment time points, intraclass correlation coefficients will be calculated for the primary outcome measures [44].

The primary analysis will focus on determining whether changes in outcome measures over time differ between the experimental groups. Repeated measures 2 × 3 ANOVA with Bonferroni correction will be used to examine interaction effects between group allocation (IMT vs. sham IMT) and time (T1, T2, T3) [44]. Additionally, we will evaluate differences between groups at each time point. Assumptions of homogeneity of variance will be assessed prior to model interpretation. Statistical significance will be set at an alpha level of 0.05 for all analyses.

## 5. Ethics and Dissemination

This study received ethical approval from the Ethics Committee of the University of West Attica (protocol number 7889-08/02/2024). No further modifications are allowed, as the study has already been revised and approved in accordance with the recommendations of the Ethics and Conduct Committee of the University of West Attica. The trial will be conducted in accordance with the Declaration of Helsinki. The confidentiality of participants will be maintained throughout the study using coded identifiers and access to data will be limited to the research team.

## 6. Protocol Amendments

### 6.1. Confidentiality

During recruitment and before they participate in the study, a confidentiality statement and a brief version of the protocol will be signed by all participants as a consent form. This will also contain a signed statement ensuring confidentiality. A code number will be obtained at the time of randomization from each participant to ensure blinding and confidentiality. Participants’ personal information will be protected throughout the study, with no disclosure of identifiable data. Authorization to publish the study’s findings will be provided upon signing the written informed consent by the participant, the principal investigator, and two witnesses, which will specify that participants may withdraw at any time without penalty.

### 6.2. Access to Data

Access to the data will be allowed only to the principal investigator, the supervisor, and the statistician.

### 6.3. Support

Professionals and members of the Physiotherapy Department at the University of West Attica committee will have overall supervision of the study process and protocol. There will be monitoring of the participants and communication from the principal investigator for four weeks after the completion of the clinical trial to address any harm or problems.

## 7. Discussion

The purpose of this randomized controlled trial is to address a significant gap in the current literature concerning the impact of inspiratory muscle training (IMT) on diaphragm functionality and symptom management in individuals with gastroesophageal reflux disease (GERD).

To date, only a limited number of studies have investigated the effectiveness of IMT on maximum inspiratory pressure, symptom severity, and quality of life in patients with GERD. However, the heterogeneity of findings makes it difficult to draw certain conclusions. Strengthening the diaphragmatic component of the antireflux barrier may contribute to symptom reduction, given that the gastroesophageal junction is anatomically supported by diaphragmatic muscle fibers [45]. Previous studies have investigated the effects of IMT on the lower esophageal sphincter (LES) and the esophagogastric junction (EGJ) pressure, with contradicting findings. Specifically, Chaves et al. (2012) found no significant alterations in LES pressure after IMT, while Souza et al. (2013), in a pre–post clinical trial, reported enhancements in EGJ function and a reduction in transient LES relaxations, with a notable improvement in GERD-related symptoms [23,46]. Furthermore, a recent systematic review evaluating the effects of inspiratory muscle training on GERD-related outcomes reported significant improvements in maximum inspiratory pressure alongside a reduction in GERDQ scores [16]. Nevertheless, the authors have emphasized several methodological limitations, including the small number of available studies, lack of randomized controlled trials, absence of power calculations for sample size determination, heterogeneity of participant characteristics, and variability in outcome measures.

We should also bear in mind the introduction of a novel IMT technique supported by electronic devices, such as the K5 system. This technique presents further advantages in training over the threshold IMT. Thus, the study may further emphasize the clinical relevance of diaphragmatic strengthening, particularly considering the documented reduction in respiratory muscle strength among individuals with GERD [46]. Additionally, the combined use of objective clinical measurements and disease-specific questionnaires is expected to provide deeper insight into the interaction between GERD and respiratory function. The present study protocol adheres to established methodological standards, including appropriate randomization procedures, concealed allocation, examiner blinding, and justified sample size calculation, thereby enhancing the internal validity and reliability of the findings.

## 8. Conclusions

This randomized controlled trial is the first to examine the impact of inspiratory muscle training with a tapered flow resistive device on maximal inspiratory pressure, peak dynamic inspiratory pressure, symptom severity, and quality of life in individuals with gastroesophageal reflux disease. Although the findings are promising, additional high-quality studies are required to further substantiate the role of IMT as a non-pharmacological approach in the management of GERD.

## Figures and Tables

**Table 1:** Standard protocol items: recommendations for interventional trials of this study (SPIRIT).

Study Procedures and Assessments	Enrollment (T-1)	Allocation (T0)	Baseline (T1)	Post-Intervention (T2)	Follow-Up (3 Months) (T3)
**Enrollment Procedures**					
Eligibility screening	X				
Informed consent obtained	X				
Collection of demographic data	X				
Inclusion of participants in study	X				
**Allocation**					
Assignment to intervention/control groups		X			
**Intervention**					
Study procedure implementation		X			
Inspiratory Muscle Training		X			
**Outcome Assessments**					
Study assessments (overall)			X	X	X
Maximal Inspiratory Pressure (MIP)			X	X	X
Maximal Expiratory Pressure (MEP)			X	X	X
5-min measurement			X	X	X
Reflux Disease Questionnaire			X	X	X
Reflux Quality of Life—Short Form (RFSF)			X	X	X
Spirometry			X	X	X

Note: X indicates that the procedure or assessment was performed at the specified time point.

## Data Availability

Data utilized and/or analyzed in this study can be obtained from the corresponding author upon reasonable request.
